# Effect of speech recognition test presentation on cochlear implant user performance

**DOI:** 10.1590/2317-1782/e20240247en

**Published:** 2026-03-30

**Authors:** Aline Faria de Sousa, Lucas Bevilacqua Alves da Costa, Rubens Vuono de Brito

**Affiliations:** 1 Alfa Instituto de Comunicação e Audição - São Paulo (SP), Brasil.; 2 Faculdade de Ciências Médicas e da Saúde, Pontifícia Universidade Católica de São Paulo – PUC-SP - São Paulo (SP), Brasil.; 3 Departamento de Otorrinolaringologia, Faculdade de Medicina, Universidade de São Paulo – USP - São Paulo (SP), Brasil.

**Keywords:** Cochlear Implants, Speech Perception, Auditory Perception, Hearing Loss, Auditory Rehabilitation

## Abstract

**Purpose:**

To analyze and compare speech recognition performance in cochlear implant (CI) users assessed with a speech recognition test administered through monitored live voice (MLV) and recorded audio, both in quiet and in noise.

**Methods:**

This cross-sectional study included 48 participants who underwent medical history assessment, audiometry, and speech recognition testing under four conditions: MLV in quiet, recorded audio in quiet, MLV in noise, and recorded audio with noise. Statistical analyses were performed using nonparametric tests, adopting a 95% confidence interval and a significance level of p < 0.05.

**Results:**

The highest performance was observed in the MLV condition in quiet, although this condition also showed greater response variability, suggesting a potential influence of evaluator-related characteristics. Performance in the MLV-in-noise and recorded-audio-in-quiet conditions was comparable, indicating that noise in live speech has an impact similar to changing the presentation mode from live to recorded speech. The lowest performance was observed in the recorded-in-noise condition, indicating that the combined effect of background noise and the absence of acoustic cues inherent to live speech negatively affects auditory performance. Word-based and sentence-based analyses produced similar outcomes.

**Conclusion:**

Speech recognition performance in CI users is influenced by both presentation mode and background noise. These findings indicate that noise may be as detrimental as the transition from live to recorded speech, underscoring the importance of standardizing speech recognition test administration.

## INTRODUCTION

Auditory habilitation and rehabilitation can be supported by several technological resources, including hearing aids, bone-anchored hearing prostheses, and cochlear implants (CI). Given the specific indication criteria for each device, comprehensive auditory assessment is recommended, incorporating electrophysiological and electroacoustic measures, audiometry, and speech recognition testing^(1)^.

Beyond device fitting, speech-language therapy focused on developing and refining auditory skills plays a central role in auditory rehabilitation^(2-4)^.

In Brazil, CI indication must adhere to Ministry of Health guidelines, which require candidates to achieve a minimum percentage of correct responses on open-set speech recognition tests. These criteria vary according to the individual’s age group (child or adult) and hearing loss onset (prelingual or postlingual). However, Ordinance No. 2,776 (2014) does not specify test administration methods—such as live voice versus recorded audio, in quiet or in noise—nor does it define scoring procedures, including whether performance should be based on correct identification of individual words or entire sentences^(5)^.

In 1996, the House Ear Institute (HEI) convened a committee to establish a minimum test battery for validating hearing device benefits and defining evaluation criteria for CI candidates. The committee emphasized test standardization and recommended an adult assessment protocol^(6)^ that included consonant–vowel–consonant (CVC) word tests for open-set word recognition^(7)^ and the Hearing in Noise Test (HINT) for open-set sentence recognition in quiet and noise^(8)^. These tests were distributed to multiple CI reference centers and provided on CD-ROM to ensure standardization and test–retest reliability across sites. Implementation of this battery coincided with technological advances that expanded access to cochlear implantation.

Over time, however, CI users began achieving ceiling-level performance on the HINT administered in quiet, prompting a revision of the minimum test battery^(9)^. The HINT was thus replaced by the AzBio Sentence Test^(10)^, which is more challenging, features multiple speakers and provides fewer contextual cues, along with the Bamford–Kowal–Bamford Speech-in-Noise (BKB-SIN) test^(11)^, designed to determine the signal-to-noise ratio required for 50% correct speech perception.

Despite these recommendations, many audiology centers in Brazil continue using live-voice speech recognition tests. A study of speech-language pathologists from 17 cochlear implant centers reported that only five used recorded audio for sentence recognition testing, with considerable variability in procedures across 10 centers, indicating a lack of standardization in test administration and selection^(12)^.

Previous studies have demonstrated that live-voice testing may overestimate speech recognition in individuals with hearing loss, with most evaluations conducted via audiometer headphones^(13,14)^.

To date, only one study has evaluated CI users in a sound-field setting while using their devices, confirming superior performance with live-voice presentation compared to recorded speech^(15)^. The authors attributed this to examiner-related factors, including pronunciation, fluency, regional accent, and fundamental frequency, which can vary over time^(16)^.

Given the need to clarify the effects of speech test presentation modes on CI user assessment, this study aimed to analyze and compare sentence recognition performance in CI users assessed via live voice versus recorded audio, and to examine the influence of noise on auditory performance.

## METHOD

This cross-sectional study was conducted between 2021 and 2023 at the Alfa Instituto de Comunicação e Audição and the Specialized Rehabilitation Center (CER III) of Hospital Universitário Alzira Velano. The sample comprised 48 cochlear implant (CI) users. The study complied with the principles of the Declaration of Helsinki of the World Medical Association (WMA) and was approved by the Research Ethics Committee of the Hospital das Clínicas, School of Medicine, University of São Paulo (HCFM/USP), Brazil (CEP/USP), under protocol number 5,900,342. Written informed consent was obtained from all participants and, when applicable, from parents or legal guardians of those under 18 years of age.

### Inclusion and exclusion criteria

Participants were initially recruited by convenience sampling and subsequently screened according to the following inclusion criteria: documented open-set speech recognition in medical records; minimum age of five years; and at least six months of CI use. Individuals with CIs who presented any neurological impairment were excluded.

### Procedures

All assessments were conducted under participants’ habitual listening conditions, reflecting their typical device use (bilateral, unilateral, or bimodal).

Speech recognition testing was administered at a fixed intensity of 60 dBA SPL in quiet. In the noise condition, the speech signal was presented at 60 dBA SPL with competing noise at 50 dBA SPL. Testing was performed in an acoustically treated booth using a two-channel digital audiometer (Interacoustic®, AC33). Speech and noise stimuli were delivered through a loudspeaker positioned at 0° azimuth in both the horizontal and vertical planes.

Participants were seated 1 m from the sound source. Sentences were presented via a notebook connected to the audiometer. Prior to testing, the output of each audiometer channel was calibrated using the pure tone included in the digital material as reference. Zero-level calibration was performed for the pure tone on channel 1 and for the noise on channel 2. Stimuli were calibrated using a digital sound level meter (Radio Shack®), set to A-weighting with fast response, positioned 1 m from the loudspeaker at 0° azimuth.

All assessments were administered by the same audiologist across all participants.

### Study procedures

Data collection included:

- **Medical history**, including age, sex, etiology of hearing loss, type of hearing loss (prelingual or postlingual), CI manufacturer, and duration of CI use. In bilateral implant users, duration of use was defined as the time elapsed since activation of the first implanted device.
- **Speech recognition testing**, conducted using the Portuguese Sentence List (Listas de Sentenças em Português – LSP)^(17)^. The LSP consists of one list of 25 sentences (List 1A) and seven additional lists of 10 sentences each (Lists 1B–7B), all phonetically balanced and containing speech-spectrum noise and a calibration pure tone. The material was studio-recorded by a male speaker. Sentence recognition was assessed in a single session using four equivalent lists (3B, 4B, 5B, and 6B), under four presentation conditions. The testing protocol was as follows:

Method 1: Monitored live-voice (MLV) presentation in quiet at 60 dBA.
Method 2: Recorded presentation in quiet at 60 dBA.
Method 3: MLV presentation in noise at a +10 dBA signal-to-noise ratio (60 dBA speech, 50 dBA noise).
Method 4: Recorded presentation in noise at a +10 dBA signal-to-noise ratio (60 dBA speech, 50 dBA noise).

For Methods 3 and 4, the competing noise was the same speech-spectrum noise contained in the sentence list recordings.

### Data analysis

Sentence recognition performance was analyzed using two approaches. The first calculated the percentage of correctly recognized words, awarding two points to each correctly repeated content word (nouns, adjectives, verbs, adverbs, and numerals) and one point for each correctly repeated function word (articles, prepositions, conjunctions, pronouns, and interjections). Scores for each list were summed and multiplied by a predefined reference value to obtain the final percentage^(18)^.

The second analysis focused on sentence-level performance, with each correctly recognized sentence corresponding to 10% of the total score for each list^(17)^.

Descriptive statistics for quantitative variables included means, medians, standard deviations, first and third quartiles, and 95% confidence intervals, as well as absolute and relative frequency distributions for qualitative variables.

### Statistical analysis

Data normality was assessed using the Kolmogorov–Smirnov and Shapiro–Wilk tests. Nonparametric statistical analyses were conducted using the Friedman test for comparisons across three or more paired conditions, followed by Wilcoxon signed-rank tests for pairwise comparisons to identify statistically significant differences between conditions.

The significance level was set at 5%. Statistical analyses were conducted using SPSS v.26, Minitab 21.2, and Microsoft Excel Office 2010.

## RESULTS

**Figure 1:**
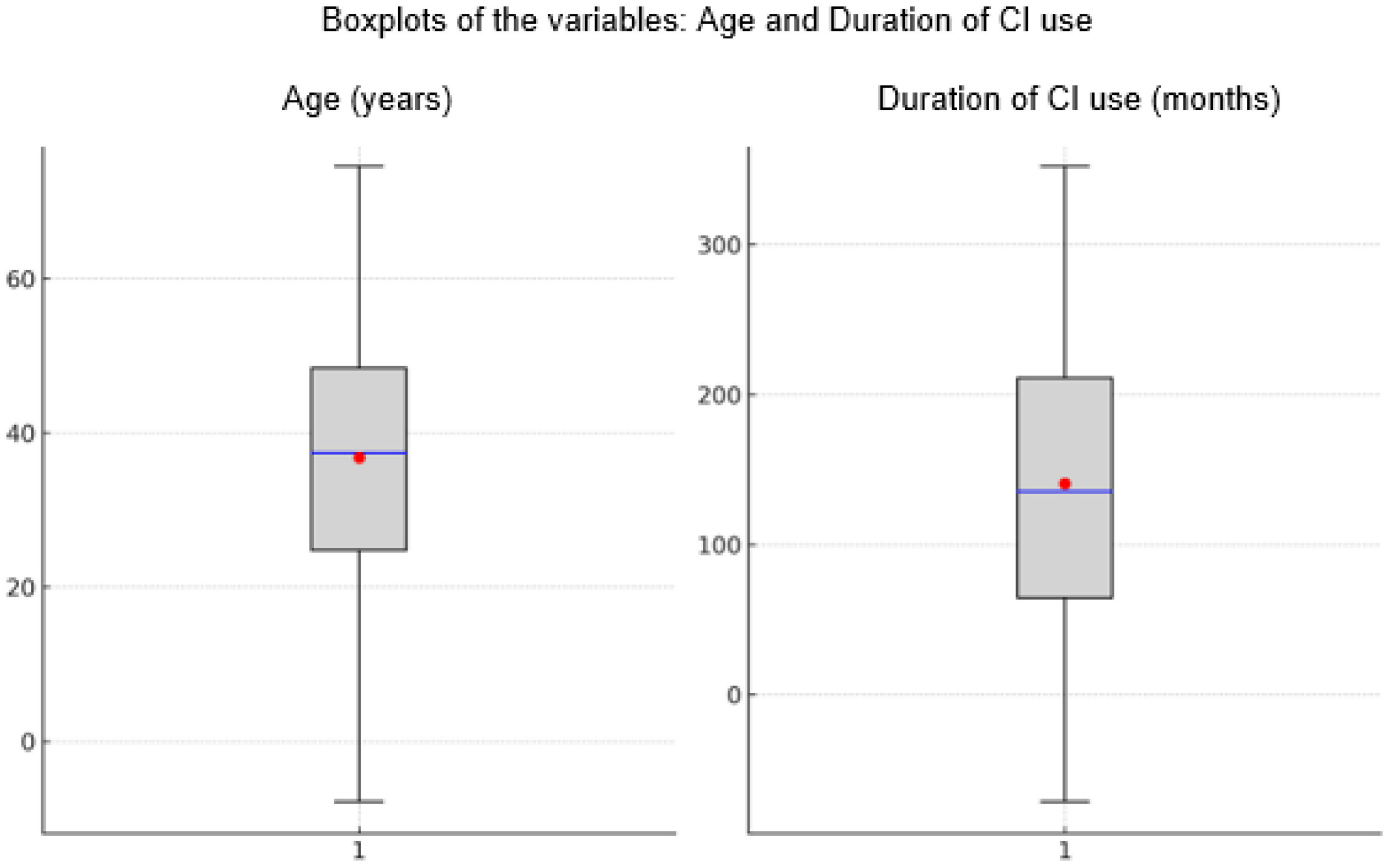
Boxplots illustrating participants’ age (years) and duration of cochlear implant (CI) use (months). The median is indicated by the blue line and the mean by the red dot

Figure 1 displays boxplots of participants’ age and duration of CI use, illustrating data distribution, including minimum and maximum values, median, quartiles, potential outliers, and means. The median is indicated by a blue line within the box, and the mean by a red dot.

Table 1 summarizes the distribution of qualitative variables, including auditory condition, etiology of hearing loss, cochlear implant brand, sex, and type of hearing loss.

**Table 1:** Distribution of qualitative characteristics

**Variable**		**n**	**%**
Auditory condition	Bilateral	20	41.67
	Bimodal	9	18.75
	Unilateral	19	39.58
Etiology of hearing loss	Idiopathic	22	45.83
	Infectious	14	29.17
	Noninfectious	12	25.00
Cochlear implant brand	AB®	7	14.58
	COCHLEAR®	23	47.92
	MED-EL®	18	37.50
Sex	Female	21	43.75
	Male	27	56.25
Type of hearing loss	Postlingual	13	27.08
	Prelingual	35	72.92

Caption: n = number of participants

Table 2 shows comparisons of speech recognition performance for words and sentences based on the percentage of correct responses.

**Table 2:** Comparison of sentence recognition test administration methods based on word-based scoring

**Scoring unit**	**Presentation mode**	**Mean**	**Median**	**Standard Deviation**	**Q1**	**Q3**	**n**	**CI**	**p-value**
Words	MLV	69.2	72.5	24.5	51.5	90.4	48	6.9	<0.001
	R	56.2	59.2	30.2	28.7	82.9	48	8.5	
	MLVN	53.1	55.4	25.3	33.6	68.7	48	7.2	
	RN	45.4	42.7	31.2	15.0	64.9	48	8.8	
Sentences	MLV	53.5	50.0	30.5	30.0	80.0	48	8.6	<0.001
	R	37.9	30.0	31.8	10.0	62.5	48	9.0	
	MLVN	38.8	40.0	27.0	20.0	52.5	48	7.6	
	RN	30.8	20.0	32.3	10.0	42.5	48	9.1	

Caption: MLV = monitored live voice; R = recorded; MLVN = monitored live voice in noise; RN = recorded in noise; Q1 = 1^st^ quartile; Q3 = 3 ^rd^ quartile; CI = confidence interval; n = total sample size; p-value = probability value

Table 3 reports the p-values from Wilcoxon pairwise comparisons. Statistically significant differences were identified between all test conditions, except between the MLV-in-noise and recorded presentation modes, for both word-based and sentence-based scoring.

**Table 3:** Post-hoc pairwise comparison p-values for word- and sentence-based scoring

**Scoring unit**	**Presentation mode**	**MLV**	**R**	**MLVN**	
Words	R	<0.001			**p-value**
	MLVN	<0.001	0.142		
	RN	<0.001	<0.001	0.003	
Sentences	R	<0.001			**p-value**
	MLVN	<0.001	0.664		
	RN	<0.001	0.02	0.012	

Caption: MLV = monitored live voice; R = recorded; MLVN = monitored live voice in noise; RN = recorded in noise; p-value = probability value

Table 4 provides descriptive data on performance differences between MLV and recorded conditions, categorized by which presentation mode yielded the better performance. Data are organized by stimulus type (words or sentences) and presence or absence of noise. Overall, most participants demonstrated superior performance with MLV presentation, particularly in quiet conditions. In the quiet condition, word-based scoring showed that 43 participants performed better under live presentation, with a mean difference of 15.58 points compared to recorded presentation. This trend persisted in the noise condition for word-based scoring (mean difference of 16.71 points) and in quiet under sentence-based scoring (mean difference of 19.07 points). By contrast, sentence-based performance in noise was similar across presentation modes, with comparable mean scores for MLV (14.59) and recorded speech (14.55).

**Table 4:** Performance differences between live-voice and recorded presentation, according to each participant’s best performance

**Best performance**			**Mean**	**Standard Deviation**	**Min**	**Max**	**n**	**CI**
Words	In quiet	MLV	15.58	12.09	0.00	52.68	43	3.61
		Recorded	9.32	12.72	2.70	32.00	5	11.15
	In noise	MLV	16.71	13.29	1.56	60.99	30	4.76
		Recorded	7.48	7.12	0.30	21.99	18	3.29
Sentences	In quiet	MLV	19.07	15.71	0.00	60.00	43	4.69
		Recorded	14.00	8.94	10.00	30.00	5	7.84
	In noise	MLV	14.59	16.60	0.00	60.00	37	5.35
		Recorded	14.55	6.88	10.00	30.00	11	4.06

Caption: MLV = monitored live voice; n = number of participants

**Figure 2:**
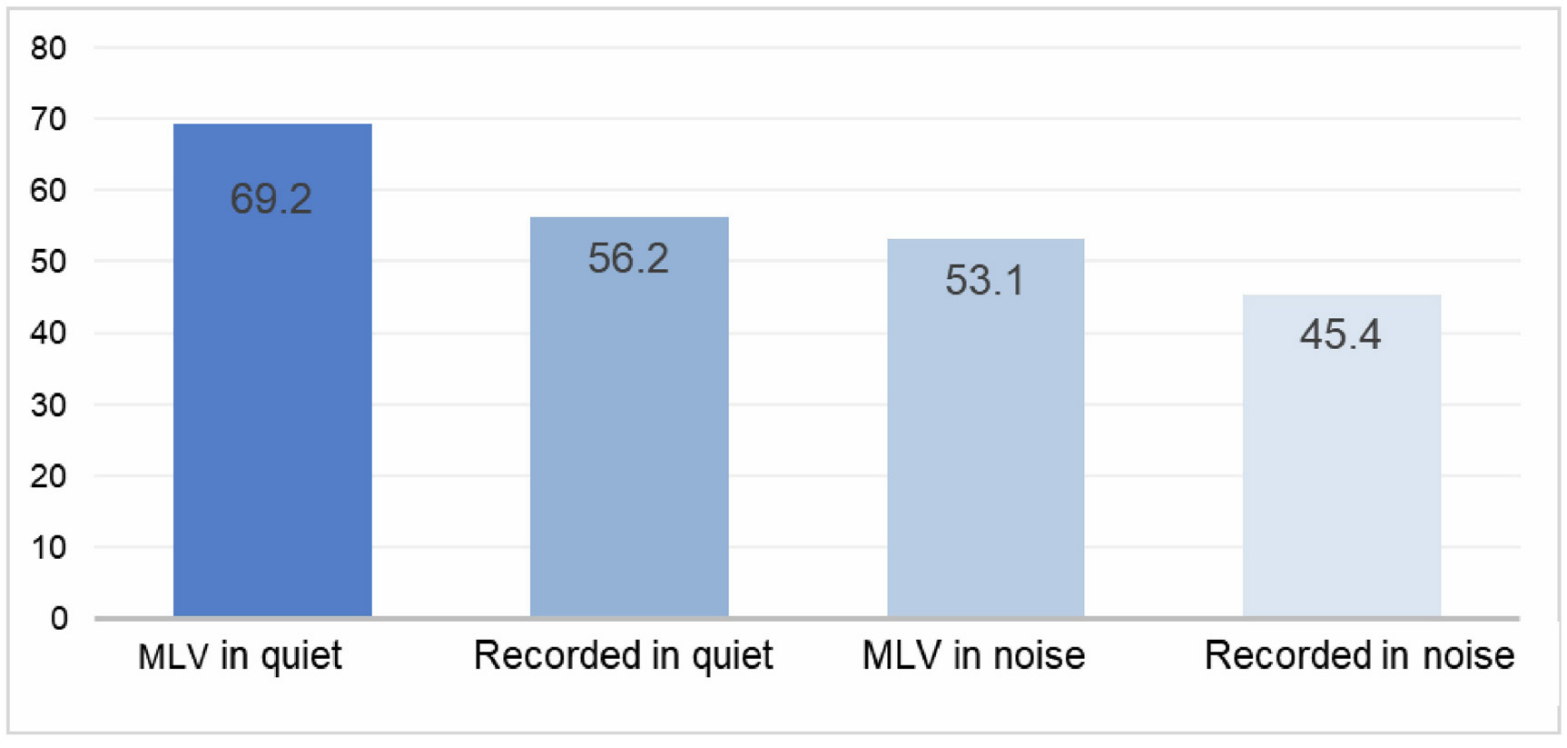
Mean percentage of correct responses by presentation mode, considering word-based scoring

**Figure 3:**
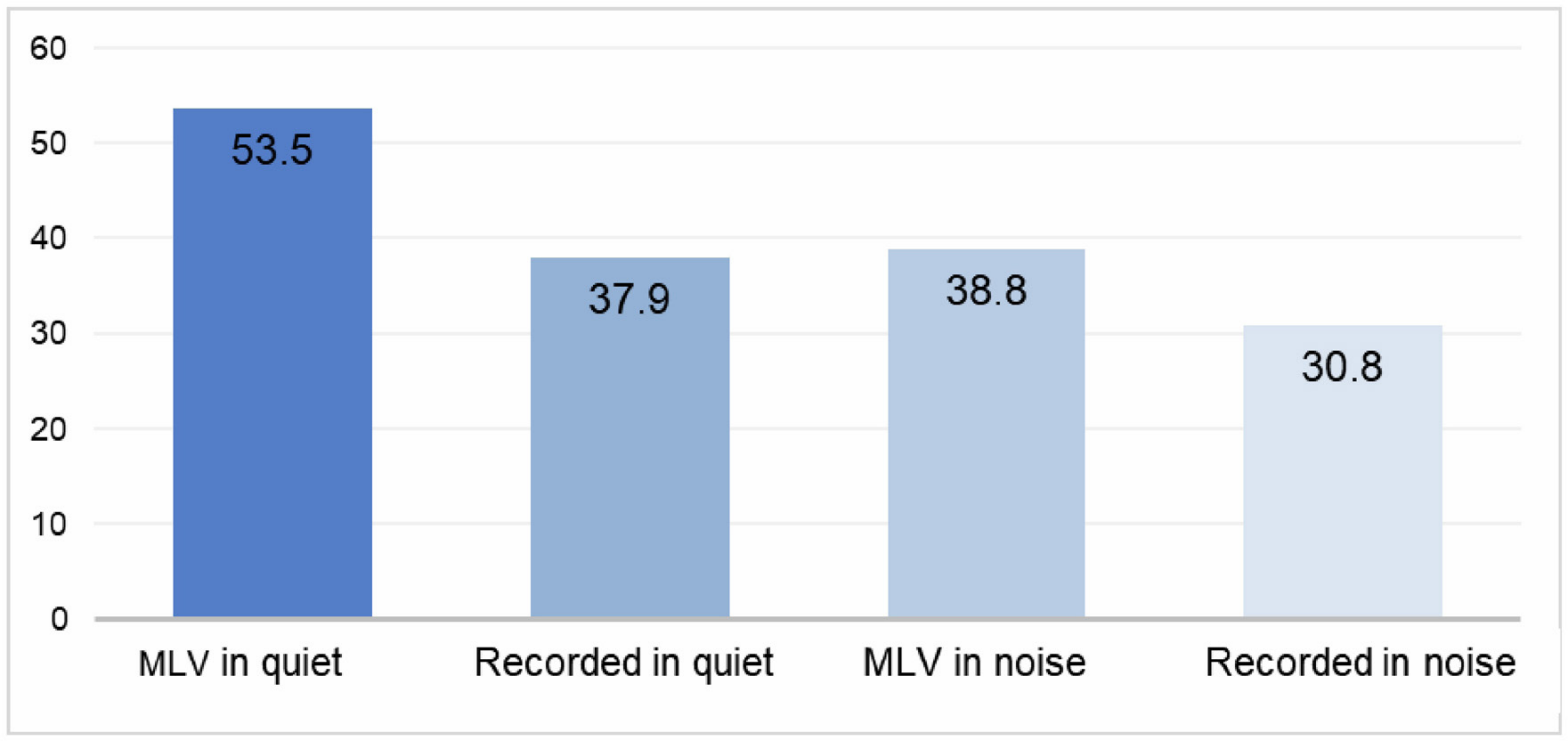
Mean percentage of correct responses by presentation mode, considering sentence-based scoring

Figures 2and 3 illustrate the mean performance of the 48 participants in the sentence recognition test based on word- and sentence-level scoring, respectively.

Caption: MLV = Monitored Live Voice

Caption: MLV = Monitored Live Voice

## DISCUSSION

In this study, speech recognition testing was conducted in a sound-field environment using both monitored live-voice and recorded presentation modes, in quiet and in noise, with participants wearing their own auditory devices. A review of the literature identified only one study directly comparing live-voice and recorded presentation in cochlear implant (CI) users. That study, conducted by Uhler et al.^(15)^, evaluated pediatric CI users and employed a presentation level of 60 dBA SPL in both conditions, consistent with the methodology used in the present investigation. The scarcity of recent studies comparing these two modes may reflect the widespread scientific consensus that recorded tests should be prioritized due to their superior standardization and reproducibility. By contrast, live-voice testing remains prevalent in clinical practice for both pediatric and adult populations, owing to its flexibility, ease of administration, and, notably, the limited availability of validated recorded speech materials in Portuguese. This clinical context, frequently observed in auditory rehabilitation services, underscores the relevance of examining how presentation mode influences speech perception outcomes. It also highlights the need to expand the availability of validated recorded materials in Portuguese to better align local clinical practice with international methodological standards.

For both word-based and sentence-based scoring, the only comparison that did not show a statistically significant difference was between MLV presentation in noise and recorded presentation in quiet. Across the remaining presentation modes, mean performance decreased progressively in the following order: MLV, recorded, MLV in noise, and recorded in noise. These results suggest that live-voice presentation in noise may impose a level of task complexity comparable to that of recorded presentation in quiet. However, this comparison should be interpreted with caution, since similar mean scores may reflect different underlying speech recognition demands. Live-voice presentation is inherently more dynamic and interactive, incorporating features such as intonation, prosody, expressiveness, and subtle articulatory variations, many of which are diminished or absent in recorded materials. The loss of these acoustic and suprasegmental cues may account for the poorer performance observed during recorded testing. Conversely, live-voice presentation is susceptible to examiner-related factors, including vocal quality, regional accent, intensity, hoarseness, and speech rate. These characteristics may vary over time due to the examiner`s health status, hormonal fluctuations, substance use, aging, and other factors, making it difficult to ensure consistent testing conditions across sessions when live voice is used^(19-21)^.

In addition, the frequent turnover of audiologists responsible for evaluating the same individual further challenges standardization. For this reason, the use of recorded speech materials is recommended whenever possible because it ensures consistent testing conditions and facilitates reliable performance comparisons over the course of auditory habilitation and/or rehabilitation. Although some studies have reported no significant differences between live-voice and recorded administration, attributing this finding to the examiner’s level of experience^(22-27)^, controlling for audiologist experience is rarely feasible in routine clinical settings, particularly in services with high staff turnover.

Based on the results of the present study, when recorded speech materials are unavailable for CI assessment, it is suggested that speech stimuli be presented with a signal-to-noise ratio of at least +10 dBA. Under these conditions, CI user performance is expected to be similar to that obtained with recorded testing in quiet. This approach, however, should be considered a last resort, reserved for exceptional circumstances in which audiology centers lack access to validated recorded speech recognition tests.

In contrast to the present findings, all participants in the study conducted by Uhler et al.^(15)^ demonstrated higher scores under live-voice presentation, with a mean improvement of 13%, ranging from 0 to 28%. In the current sample, most participants also performed better with MLV, regardless of noise. When performance was analyzed using word-based scoring in quiet conditions, the mean difference was 15%, ranging from 0 to 52.68%.

Although fewer in number, some participants performed better in recorded testing, consistent with findings reported in two previous studies^(16,27)^. In word-based scoring, this subgroup exhibited lower score variability, suggesting that the minor, non-significant variations in recorded-test scores may reflect expected test–retest variability rather than systematic effects of presentation mode.

In noisy conditions, while some participants scored higher with recorded presentation, both the standard deviation and mean difference were smaller than those observed in all other presentation modes using word-based scoring. These findings indicate that increasing task complexity is associated with reduced variability in participant performance. Moreover, word-based scoring appears to provide more sensitive data for examining these differences. Accordingly, CI users should be assessed using both word- and sentence-based scoring. As noted by Martin^(28)^, sentence comprehension requires not only auditory skills but also cognitive, mnemonic, and auditory processing abilities. This is largely due to the greater linguistic redundancy inherent in sentence-level stimuli. Conversely, analyses focused on words and/or phonemes tend to more effectively capture auditory skill performance, since these stimuli are less redundant. Consequently, word- and sentence-based scoring approaches yield distinct yet complementary information that should be integrated into the comprehensive evaluation of CI users.

Given the influence of uncontrollable variables inherent to live-voice speech recognition testing, we recommend the use of recorded materials to ensure consistent testing conditions across test–retest scenarios. Furthermore, because the choice of scoring method (words or sentences) may influence interpretation of CI user benefits in relation to device mapping, signal-processing strategies, and therapeutic monitoring, audiologists should systematically analyze both approaches.

In summary, the present study demonstrates that speech recognition performance in CI users varies by test presentation mode, except when comparing live-voice presentation in noise with recorded presentation in quiet. Superior performance was observed with live-voice presentation in all other comparisons. With respect to scoring strategies, word-based scoring appears to be more sensitive in identifying differences between testing conditions
