# Supplementary material for: Effect of speech recognition test presentation on cochlear implant user performance
Source: Codas. 2026 Mar 30;38(2):e20240247. doi: 10.1590/2317-1782/e20240247en (PMC13075849; doi:10.1590/2317-1782/e20240247en)
Supplement: Translation

# Translation: Influência da forma de aplicação de testes de reconhecimento de fala no desempenho de usuários de implante coclear

**DOI:** 10.1590/2317-1782/e20240247pt

**Authors:** Aline Faria de Sousa, Lucas Bevilacqua Alves da Costa, Rubens Vuono de Brito

**Affiliations:** 1 Alfa Instituto de Comunicação e Audição - São Paulo (SP), Brasil.; 2 Faculdade de Ciências Médicas e da Saúde, Pontifícia Universidade Católica de São Paulo – PUC-SP - São Paulo (SP), Brasil.; 3 Departamento de Otorrinolaringologia, Faculdade de Medicina, Universidade de São Paulo – USP - São Paulo (SP), Brasil.

**Keywords:** Implantes Cocleares, Percepção da Fala, Percepção Auditiva, Perda Auditiva, Reabilitação

## Abstract

**Objetivo:**

Analisar e comparar o desempenho no reconhecimento de fala de usuários de implante coclear (IC) por meio do teste de reconhecimento de sentenças aplicado à viva voz e por áudio gravado, com e sem ruído.

**Método:**

Estudo transversal com 48 participantes, avaliados por anamnese, audiometria e teste de reconhecimento de fala em quatro condições: viva voz, áudio gravado, viva voz com ruído e áudio gravado com ruído. A análise estatística utilizou testes não paramétricos, com intervalo de confiança de 95% e nível de significância de p<0,05.

**Resultados:**

O melhor desempenho foi observado na condição viva voz sem ruído, com maior variabilidade nas respostas, sugerindo possível influência das características do avaliador. O desempenho nas condições viva voz com ruído e áudio gravado sem ruído foi semelhante, indicando que o ruído na fala ao vivo impacta tanto quanto a troca do modo de apresentação de viva voz para gravado. A pior performance foi na condição áudio gravado com ruído, evidenciando que a combinação do ruído com a ausência dos elementos acústicos da fala ao vivo impacta negativamente a performance auditiva. As análises por palavras e por sentenças foram semelhantes.

**Conclusão:**

O desempenho de usuários de IC no reconhecimento de fala é afetado tanto pela forma de apresentação quanto pela presença de ruído. Os resultados reforçam que o ruído pode ter efeito tão impactante quanto a mudança da fala ao vivo para gravada, destacando a necessidade de padronização na aplicação dos testes.

## INTRODUÇÃO

A habilitação e reabilitação auditiva podem ser realizadas por meio de tecnologias disponíveis como os aparelhos de amplificação sonora individual (AASI), as próteses osteancoradas ao osso (POAO) e o implante coclear (IC). Existem critérios particulares para a recomendação de cada dispositivo e por isso, é sugerida a realização de uma análise auditiva abrangente através de procedimentos eletrofisiológicos, eletroacústicos, audiometria e testes de reconhecimento da fala^(1)^

Além do uso do dispositivo é importante ressaltar a importância da fonoterapia voltada ao desenvolvimento e aperfeiçoamento das habilidades auditivas no processo de habilitação e reabilitação auditiva^(2-4)^.

No Brasil a indicação do IC deve respeitar as diretrizes Ministério da Saúde, que estabelece que o candidato deve apresentar uma determinada porcentagem de acertos nos testes de reconhecimento da fala em conjunto aberto, cujo critério varia de acordo com as características do indivíduo (criança ou adulto) e do ponto de início da perda auditiva (pré ou pós lingual). Contudo, a Portaria n. 2.776 (2014) não menciona a forma com que o teste deve ser realizado, se à viva voz, com áudio gravado, com ruído ou sem ruído, nem como a pontuação dos testes deve ser calculada, considerando o acerto de palavras individuais na sentença ou a sentença completa^(5)^.

Em 1996 o House Ear Institute (HEI) realizou um comitê para instituir uma bateria mínima de testes com o objetivo de validar o benefício do uso dos dispositivos auditivos, além de detalhar os critérios de avaliação dos candidatos ao IC. Para tanto, o comitê enfatizou que os testes deveriam ser padronizados e sugeriu o uso do seguinte protocolo para a avaliação de adultos^(6)^: para avaliação de palavras em conjunto aberto, aplica-se o teste com palavras no formato consoante-vogal-consoante^(7)^ e para a avaliação do reconhecimento de sentenças em conjunto aberto no silêncio e no ruído, utiliza-se o teste Hearing in Noise Test (HINT)^(8)^. Todos os testes foram distribuídos para diversos centros de referência em IC e disponibilizados por meio de gravação em CD ROMs, para garantir a padronização e validade dos mesmos em situações de teste e reteste em diferentes localidades. Após o estabelecimento da bateria mínima de avaliação, houve um grande avanço na tecnologia e mais pessoas receberam o dispositivo.

Entretanto, os usuários de IC passaram a alcançar a pontuação máxima no HINT apresentado em silêncio, devido ao efeito de teto observado para o teste, tornou-se necessária a revisão da bateria mínima de testes^(9)^, sendo então substituído o teste de sentenças *HINT* pelos teste Sentenças AzBio^(10)^, por ser considerado um teste mais complexo e apresentar diferentes vozes e menos pistas contextuais e, o teste *Bamford-Kowal-Bamford Speech-in-Noise (BKBSIN)* ^(11)^, que avalia a relação sinal ruído necessária para que o candidato alcance 50% de acertos na percepção auditiva da fala.

Muitos centros de audiologia no Brasil realizam os testes de reconhecimento da fala à viva voz. Um estudo constatou a partir dos profissionais fonoaudiólogos inseridos em dezessete diferentes centros de implante coclear, que apenas 5 serviços realizavam testes de reconhecimento de sentenças com áudio gravado. Também foi identificada variabilidade dos testes em dez centros, demonstrando que não há padronização em relação à metodologia de aplicação e ao modelo do teste utilizado^(12)^.

Outros estudos evidenciaram que os testes à viva voz podem superestimar o desempenho dos indivíduos com deficiência auditiva, na maior parte dos estudos os testes foram realizados por meio de fones de ouvido do audiômetro^(13,14)^.

Apenas um estudo realizou os testes em campo sonoro com usuários de IC, estes estavam utilizando seus dispositivos auditivos no momento dos testes. Confirmou-se que os usuários de IC apresentam melhor desempenho no teste à viva voz do que no teste gravado^(15)^. Os pesquisadores atribuem este efeito a possíveis variáveis relacionadas às características vocais do examinador, tais como: pronúncia, fluência, acento regional e frequência fundamental, que podem variar ao longo do tempo^(16)^.

Tendo em vista a necessidade de entender o impacto das duas formas de aplicação (viva voz *versus* gravado) dos testes de reconhecimento da fala para avaliação usuários de IC, esta pesquisa teve como objetivo analisar e comparar o desempenho de reconhecimento da fala de usuários de implante coclear por meio do teste de reconhecimento de sentenças aplicado de forma à viva voz e áudio gravado e determinar a influência do ruído na performance auditiva

## MÉTODO

Trata-se de um estudo transversal, realizado no Alfa Instituto de Comunicação e Audição e no Centro Especializado em Reabilitação (CER III) do Hospital Universitário Alzira Velano. A pesquisa foi desenvolvida entre os anos de 2021 e 2023, incluiu 48 participantes usuários de IC e atendeu aos princípios éticos para a investigação científica envolvendo seres humanos, estabelecidos pela Declaration of Helsinki of the World Medical Association (WMA). O estudo foi aprovado por Comitê de Ética em Pesquisa do Hospital das Clínicas da Faculdade de Medicina da Universidade de São Paulo (HCFM/USP), Brasil (CEP/USP), sob parecer de número 5.900.342 e obteve o consentimento informado por todos os participantes do estudo, bem como o consentimento informado de seus pais quando os participantes tinham idade menor que 18 anos.

### Critérios de inclusão e exclusão

A amostra foi selecionada inicialmente por conveniência e em seguida avaliada para o atendimento dos seguintes critérios: Apresentar reconhecimento da fala em conjunto aberto com dados registrados em prontuário; Idade mínima de cinco anos; Tempo mínimo de seis meses de utilização do IC. Foram desconsiderados os usuários de IC com qualquer tipo de comprometimento neurológico.

### Procedimentos

Todos os testes foram realizados na condição auditiva habitual do paciente, ou seja, da forma que ele costuma utilizar seus dispositivos (bilateral, unilateral ou bimodal).

Os testes utilizaram intensidade fixa de 60 dBA NPS na condição sem ruído e 60 dBA NPS de sinal de fala e 50dBA NPS de ruído na condição com ruído, e foram realizados em cabine acusticamente tratada com audiômetro digital de dois canais, marca Interacoustic®, modelo AC33. A incidência do sinal de fala e do ruído foi em 0º azimute nos planos horizontal e vertical.

Os sujeitos foram posicionados a 1 m de distância da fonte sonora. As sentenças foram apresentadas com auxílio de um notebook acoplado ao audiômetro. A saída de cada canal do “Volume Units Meter” (VU-meter) do audiômetro foi previamente calibrada, utilizando-se como referência o tom puro presente no material digital. Houve também a calibração a nível zero para o tom puro presente no canal 01 e para o ruído presente no canal 02. O estímulo foi calibrado utilizando como referência a escala A (resposta rápida) de um decibelímetro digital da marca Radio Shack®, e o equipamento foi posicionado à um metro de distância da caixa a 0 grau azimute.

Todos os testes foram aplicados pela mesma audiologista em todos os participantes.

### Dados do estudo

Foram realizados os seguintes procedimentos:

- Anamnese para coleta de dados como: idade; sexo; etiologia da perda auditiva; tipo de surdez (pré ou pós lingual); marca do implante utilizado; tempo de uso do IC, sendo considerando para os usuários bilaterais o tempo de uso do IC mais antigo.
- Testes de Reconhecimento de Fala: Foi aplicada a Listas de Sentenças em Português (LSP)^(17)^. O teste LSP é composto por uma lista de 25 sentenças denominada Lista 1A e outras sete listas contendo 10 sentenças cada (1B a 7B). Todas as listas foram balanceadas foneticamente e apresentaram ruído de espectro de fala e um tom puro de calibração, sendo gravadas em estúdio por um locutor com voz masculina. Os participantes realizaram os testes de sentenças na mesma sessão e com a utilização das listas 3B, 4B, 5B e 6B e quatro métodos diferentes de aplicação, é importante ressaltar que as Listas são compatíveis entre si no que diz respeito ao nível de dificuldade. Portanto, seguiu-se o cronograma abaixo:

Método 1: Apresentação do teste à viva voz sem ruído a 60 dBA.
Método 2: Apresentação do teste gravado sem ruído a 60 dBA.
Método 3: Apresentação do teste à viva voz com relação sinal/ruído de +10 dBA, utilizando 60 dBA para o sinal de fala e 50 dBA para o ruído.
Método 4: Apresentação do teste gravado com relação sinal/ruído de +10 dBA, sendo utilizados 60 dBA para o sinal de fala e 50 dBA para o ruído.

Para os métodos 3 e 4 nos quais o som competitivo esteve presente, houve a padronização do procedimento através da utilização do mesmo ruído disponível na gravação da lista de sentenças (ruído de espectro de fala).

### Análise dos dados

Para o teste de sentenças foram realizadas duas análises. A primeira considerou o percentual de acertos de palavras, atribuindo dois pontos para cada palavra de conteúdo (substantivos, adjetivos, verbos, advérbios e numerais) e um ponto para cada palavra funcional (artigos, preposições, conjunções, pronomes e interjeições), repetida corretamente. Ao final da apresentação da lista os pontos foram somados e multiplicados por um valor de referência pré-estabelecido, para obter o percentual final de acertos^(18).^

A segunda análise esteve orientada para o cálculo do percentual de acertos de sentenças, sendo atribuídos 10% para cada sentença de cada lista^(17)^.

A análise descritiva apresentou as médias, medianas, desvios padrão, 1º quartis, 3º quartis e intervalos de confiança para as variáveis quantitativas e as distribuições de frequência absoluta e relativa para as variáveis qualitativas.

### Análise estatística

Foram aplicados os testes Kolmogorov-Smirnov e Shapiro Wilk para determinar a normalidade dos dados, seguido dos testes não-paramétricos de *Friedman para comparação dos testes* (comparação de dados pareados de 3 ou mais variáveis) e *Wilcoxon* para comparação por pares, para determinar entre quais testes exatamente ocorreu a diferença significativa.

O nível de significância adotado foi de 5% e para as análises foram utilizados os softwares SPSS v.26, Minitab 21.2 e Excel Office 2010.

## RESULTADOS

A Figura 1 apresenta os boxplots referentes à idade dos participantes e ao tempo de uso do implante coclear, possibilitando a visualização da distribuição dos dados, incluindo valores mínimos e máximos, mediana, quartis, possíveis outliers e a média. A mediana é representada pela linha azul no interior da caixa, enquanto a média é indicada por um ponto vermelho.

**Figura 1:** Boxplots das variáveis idade (em anos) e tempo de uso do implante coclear (em meses), com indicação da mediana (linha azul) e da média (ponto vermelho)

Na Tabela 1 encontra-se a distribuição das seguintes variáveis qualitativas: condição auditiva, etiologia da perda auditiva, marca do IC, sexo e tipo de surdez.

**Tabela 1:** Distribuição das variáveis qualitativas

**Variáveis**		**n**	**%**
Condição Auditiva	Bilateral	20	41,67
	Bimodal	9	18,75
	Unilateral	19	39,58
Etiologia da Perda Auditiva	Idiopática	22	45,83
	Infecciosa	14	29,17
	Não infecciosa	12	25,00
Marca do IC	AB®	7	14,58
	COCHLEAR®	23	47,92
	MEDEL®	18	37,50
Sexo	Feminino	21	43,75
	Masculino	27	56,25
Tipo de surdez	Pós-lingual	13	27,08
	Pré-lingual	35	72,92

Legenda: n = número de participantes

Na Tabela 2 estão apresentadas as comparações dos resultados dos testes considerando o percentual de acertos de palavras e sentenças.

**Tabela 2:** Comparação dos métodos de aplicação dos testes de reconhecimento de sentenças considerando a pontuação por palavras

**Pontuação**	**Modalidade**	**Média**	**Mediana**	**Desvio Padrão**	**Q1**	**Q3**	**n**	**IC**	**p-valor**
Palavras	VV	69,2	72,5	24,5	51,5	90,4	48	6,9	<0,001
	G	56,2	59,2	30,2	28,7	82,9	48	8,5	
	VVR	53,1	55,4	25,3	33,6	68,7	48	7,2	
	GR	45,4	42,7	31,2	15,0	64,9	48	8,8	
Sentenças	VV	53,5	50,0	30,5	30,0	80,0	48	8,6	<0,001
	G	37,9	30,0	31,8	10,0	62,5	48	9,0	
	VVR	38,8	40,0	27,0	20,0	52,5	48	7,6	
	GR	30,8	20,0	32,3	10,0	42,5	48	9,1	

Legenda: VV = Viva voz; G = gravado; VVR = Viva voz com ruído; GR = gravado com ruído; Q1 = 1º quartil; Q3 = 3º quartil; IC = intervalo de confiança; n = número total de participantes; p-valor = valor de probabilidade

Na Tabela 3 observa-se os p-valores (pelo teste de Wilcoxon) das comparações entre os testes aos pares. Houve diferença estatisticamente significante entre todos os testes, exceto entre os testes viva voz no ruído e gravado, tanto para a pontuação por palavras como para sentenças.

**Tabela 3:** P-valores do post-hoc da tabela 3 para pontuação por palavras e sentenças

**Pontuação**	**Modalidade**	**VV**	**G**	**VVR**	
Palavras	G	<0,001			**p-valor**
	VVR	<0,001	0,142		
	GR	<0,001	<0,001	0,003	
Sentenças	G	<0,001			**p-valor**
	VVR	<0,001	0,664		
	GR	<0,001	0,02	0,012	

Legenda: VV = Viva voz; G = gravado; VVR = Viva voz com ruído; GR = gravado com ruído; p-valor = valor de probabilidade

A Tabela 4 apresenta os dados descritivos da diferença de desempenho entre os testes aplicados em viva voz e gravado, considerando o modo de apresentação em que cada participante obteve melhor resultado. Os dados estão organizados por tipo de estímulo (palavras ou sentenças), com e sem presença de ruído. Observa-se que, de modo geral, a maioria dos participantes teve melhor desempenho no modo viva voz, especialmente nas condições sem ruído. Para palavras sem ruído, 43 participantes apresentaram melhor desempenho em viva voz, com uma diferença média de 15,58 pontos em relação ao modo gravado. Essa tendência se manteve em palavras com ruído (diferença média de 16,71 pontos) e em sentenças sem ruído (diferença média de 19,07 pontos). Na condição de sentenças com ruído, os resultados foram semelhantes entre os modos, com médias próximas para viva voz (14,59) e gravado (14,55).

**Tabela 4:** Diferença de desempenho entre os modos de apresentação (viva voz e gravado), conforme o melhor resultado individual

**Melhor desempenho na Apresentação**			**Média**	**Desvio Padrão**	**Min**	**Max**	**n**	**IC**
Palavras	Sem ruído	Viva Voz	15,58	12,09	0,00	52,68	43	3,61
		Gravado	9,32	12,72	2,70	32,00	5	11,15
	Ruído	Viva Voz	16,71	13,29	1,56	60,99	30	4,76
		Gravado	7,48	7,12	0,30	21,99	18	3,29
Sentenças	Sem ruído	Viva Voz	19,07	15,71	0,00	60,00	43	4,69
		Gravado	14,00	8,94	10,00	30,00	5	7,84
	Ruído	Viva Voz	14,59	16,60	0,00	60,00	37	5,35
		Gravado	14,55	6,88	10,00	30,00	11	4,06

Legenda: n = número de participantes

Na Figura 2 é apresentada a média do desempenho dos 48 participantes que realizaram o teste de sentenças, considerando a pontuação por palavras.

**Figura 2:** Percentual médio de acertos de acordo com a forma de apresentação e considerando a pontuação por palavras

Na Figura 3 é apresentada a média do desempenho dos 48 participantes considerando a pontuação por sentenças.

**Figura 3:** Percentual médio de acertos de acordo com a forma de apresentação e considerando a pontuação por sentenças

## DISCUSSÃO

Neste estudo, os testes foram aplicados em campo, apresentados à viva voz e por áudio gravado, com e sem ruído, e os participantes estavam utilizando seus dispositivos auditivos. Na literatura, foi encontrado apenas um estudo que investigou as diferenças entre as formas de apresentação à viva voz e gravado em usuários de implante coclear, realizado por Uhler et al.^(15)^, com crianças usuárias de IC, utilizando intensidade de 60 dBA NPS (A) em ambas as condições, assim como no presente estudo. A escassez de publicações atuais que comparam essas duas formas de apresentação pode estar relacionada ao fato de que, no contexto científico, há um consenso de que os testes gravados devem ser priorizados, por garantirem maior padronização e reprodutibilidade dos resultados. Contudo, na prática clínica, a aplicação à viva voz ainda é amplamente utilizada, tanto em avaliações pediátricas quanto adultas, devido à sua flexibilidade, facilidade de execução e, especialmente, à limitada disponibilidade de materiais gravados validados em língua portuguesa. Essa realidade, frequentemente observada nos serviços de reabilitação auditiva, reforça a importância de investigar o impacto que a forma de apresentação pode ter nos resultados dos testes de percepção de fala. Além disso, evidencia a necessidade de ampliar a oferta de materiais gravados em português, promovendo maior alinhamento entre a prática clínica e os padrões metodológicos preconizados pela literatura científica internacional.

Nesta pesquisa, constatou-se que considerando-se a pontuação por palavras e sentenças só não houve diferença estatisticamente significante entre as apresentações na modalidade à viva voz na presença de ruído e gravada sem ruído. Em relação às demais formas de apresentação, os indivíduos tiveram média de acertos maior nas seguintes formas de apresentação, respectivamente: à viva voz, gravado, viva voz no ruído e gravado no ruído. Tal resultado nos sugere que a complexidade da forma de apresentação à viva voz na presença de ruído seria equivalente à forma gravada sem o ruído. No entanto, essa comparação demanda cautela, pois embora os escores médios sejam semelhantes, trata-se de tarefas que envolvem habilidades distintas de reconhecimento de fala. A apresentação à viva voz, por ser mais dinâmica, natural e interativa, envolve características como entonação, prosódia, expressividade e microvariações articulatórias, elementos que são, em grande parte, perdidos nas versões gravadas. A perda desses elementos em uma gravação pode justificar um desempenho pior no teste gravado. No entanto, as apresentações à viva voz podem ser influenciadas por fatores como o timbre vocal, sotaque, intensidade, rouquidão e velocidade do estímulo, e esses fatores podem variar ao longo do tempo devido a questões de saúde, variações hormonais, uso de substâncias, idade e outras situações, tornando inviável a manutenção das mesmas condições em diferentes ocasiões na aplicação do teste à viva voz^(19-21)^.

A rotatividade frequente de audiologistas que podem avaliar o mesmo indivíduo também torna a padronização desafiadora. Portanto, é aconselhável que o teste seja sempre realizado com gravações de áudio, mantendo condições consistentes para permitir a comparação do desempenho do indivíduo ao longo de seu processo de habilitação e/ou reabilitação auditiva. Alguns estudos não encontraram diferenças significativas entre as formas de aplicação à viva voz e gravada, estes estudos sugerem que essa ausência de diferenças seja decorrente da experiência do audiologista ao aplicar o teste^(22-27)^

Conforme mencionado, em muitos serviços há alta rotatividade de profissionais e controlar a variável experiência do audiologista é certamente uma tarefa inviável.

No entanto, levando-se em consideração os resultados do presente estudo, se não for possível usar gravações de áudio ao se avaliar usuários de IC, sugere-se que o audiologista apresente o estímulo de fala com pelo menos 10 dBA de relação sinal/ruído, já que em tal condição espera-se que o desempenho do usuário de IC seja próximo do que seria obtido em um teste gravado sem ruído. Tal situação não é a ideal e só deve ser considerada em situações extremas, quando o centro de audiologia não tiver acesso à testes de reconhecimento de fala gravados.

Diferente do presente estudo, todos os participantes da pesquisa conduzida por Uhler et al.^(15)^, pontuaram mais nas apresentações à viva voz, sendo em média 13% melhor e com variação de 0 a 28%. Em relação a maioria dos participantes, houve melhor desempenho na apresentação à viva voz independente da presença do ruído. Na condição sem ruído houve uma diferença média de 15% com variação de 0 até 52,68% considerando a pontuação por palavras.

Embora minoria, alguns participantes tiveram melhor desempenho nos testes gravados, o que corrobora com outros dois estudos^(16,27)^. Ao se considerar a pontuação por palavras, é possível notar menor variabilidade dos resultados desta minoria. Este achado nos sugere que a variação dos escores na forma gravada, por ser menor e não ter significância estatística, pode ser explicada por uma variação esperada de teste-reteste.

Na condição com ruído, houve participantes com melhor performance na apresentação gravada, no entanto, o desvio padrão e a diferença média dos resultados foi menor que em todas as formas de apresentação quando considerada a pontuação por palavras. Observa-se, portanto, que conforme há maior complexidade na forma de apresentação do teste, há menor variabilidade nos resultados dos participantes. Além disso, é importante ressaltar que a pontuação por palavras nos fornece dados mais relevantes ao se analisar tais diferenças. Sendo assim, ao se avaliar usuários de IC recomenda-se que tanto a pontuação por palavras como por sentenças seja considerada pela equipe. Martin^(28)^ refere que para a compreensão de sentenças pelo indivíduo, são exigidas habilidades auditivas e outras, que estão associadas à cognição, memória e processamento auditivo. Isso se deve à maior redundância do estímulo, ao contrário, quando levamos em consideração as palavras e/ou fonemas tendemos a avaliar melhor os aspectos relacionados às habilidades auditivas, pois o conteúdo é menos redundante. As duas formas de pontuar o teste nos traz informações diferentes e complementares que devem ser consideradas na avaliação global do usuário de IC.

Portanto, devido à influência de variáveis que não podem ser controladas ao se apresentar um teste de reconhecimento de fala na modalidade à viva voz, recomenda-se que o fonoaudiólogo apresente o teste na forma gravada, mantendo assim, as mesmas condições em situações de teste-reteste. Além disso, tendo em vista que a escolha da pontuação (sentenças ou palavras) poderá impactar na análise do benefício que o usuário de IC possui, seja esta realizada em relação à um novo mapeamento, uso de alguma estratégia específica de processamento, e até mesmo o monitoramento da evolução terapêutica com fonoterapia, recomenda-se que o audiologista analise as duas formas de pontuação.

Sendo assim, este estudo conclui que há diferença na performance de reconhecimento da fala por usuários de IC, entre as formas de apresentação do teste, exceto para as apresentações à viva voz com ruído e gravado sem ruído. Nas demais avaliações houve melhor performance quando o teste foi apresentado à viva voz. Quanto à comparação das formas de se pontuar o teste, a pontuação por palavras tende a ser mais sensível na verificação das diferenças entre os testes.
